# Machine Learning for Medical Applications

**DOI:** 10.1155/2015/825267

**Published:** 2015-01-26

**Authors:** Huiyu Zhou, Jinshan Tang, Huiru Zheng

**Affiliations:** ^1^School of Electronics, Electrical Engineering and Computer Science, The Queen's University of Belfast, Belfast BT9 6AZ, UK; ^2^School of Technology, Michigan Technological University, Houghton, MI 49931, USA; ^3^School of Computing and Mathematics, University of Ulster, Jordanstown Campus, Shore Road, Newtownabbey BT37 0QB, UK

Machine learning (ML) has been well recognized as an effective tool for researchers to handle the problems in signal and image processing. Machine learning is capable of offering automatic learning techniques to excerpt common patterns from empirical data and then make sophisticated decisions, based on the learned behaviors. Medicine has a large dimensionality of data and the medical application problems frequently make the human-generated, rule-based heuristics intractable. In this special issue, we provide a forum to present the cutting-edge machine learning techniques in medical applications, including the learning of similarities across different image modalities, organ localization, learning of anatomical changes, tissue classification, and computer-aided diagnosis.

The topics of the accepted papers in this Special Issue spread from electroencephalography (EEG) signal processing to image segmentation. Z. Yang et al. in* “Adaptive neuro-fuzzy inference system for classification of background EEG signals from ESES patients and controls”* introduced an adaptive neurofuzzy inference system for classification of background EEG signals from the patients of slow-wave sleep syndrome and control subjects. Their study showed that the entropy measures of EEG were significantly different between the patients and normal subjects. Therefore, a classification framework based on entropy measures was proposed. S. Jirayucharoensak et al. in* “EEG-based emotion recognition using deep learning network with principal component based covariate shift adaptation”* proposed the utilization of a deep learning network (DLN) to discover unknown feature correlation between input signals. The DLN was implemented with a stacked autoencoder (SAE) using hierarchical feature learning approach. D. Al-Jumeily et al. in* “A novel method of early diagnosis of Alzheimer's disease based on EEG signals”* introduced three neural synchrony measurement techniques: phase synchrony, magnitude squared coherence, and cross correlation for classification of mild Alzheimer's disease patients and healthy subjects. K. Zhang et al. in* “Adaptive bacteria colony picking in unstructured environments using intensity histogram and unascertained LS-SVM classifier”* presented a novel approach for adaptive colony segmentation in unstructured environments by treating the detected peaks of intensity histograms as a morphological feature of images. In order to avoid disturbing peaks, an entropy based mean shift filter was introduced to smooth images as a preprocessing step. The relevance and importance of these features can be determined in an improved support vector machine classifier using unascertained least square estimation. M. Cabrerizo et al. in* “Induced effects of transcranial magnetic stimulation on the autonomic nervous system and the cardiac rhythm”* demonstrated that repetitive transcranial magnetic stimulation (rTMS) could induce changes in the heart rhythm.

